# Mentorship Scheme – a Novel Approach for Plugging the Gap in Differential Attainment for Psychiatry Core Trainees in East Midlands

**DOI:** 10.1192/bjo.2025.10303

**Published:** 2025-06-20

**Authors:** Samreen Samad, Awhangansi Sewanu, Champion Seun-Fadipe, Nick Long, Ian Yanson

**Affiliations:** 1 Leicestershire Partnership NHS trust, Leicester, United Kingdom; 2 Nottinghamshire Healthcare NHS Foundation Trust, Nottingham, United Kingdom; 3 Derbyshire Healthcare NHS Foundation Trust, Derby, United Kingdom

## Abstract

**Aims:** The MRCPsych results report and GMC annual report on trainee performance highlighted that UK PMQ candidates perform better than OS PMQ candidates and that White candidates perform better compared with candidates with other ethnic backgrounds. A mentoring scheme was designed as a proposed solution to bridge the gap of differential attainment in Core trainees in Psychiatry with a focus on improving ARCP outcome and Exam Pass rate in Psychiatry.

**Methods:** The Mentorship Scheme was piloted between August 2023 to August 2024 among Core trainees and Higher Trainees working in Psychiatry in Mental Health Trusts in East Midlands. Higher trainees took part in the project as mentors and were required to complete mentorship course from e-lfh hub prior to start of mentorship.

The evaluation was of a longitudinal, prospective design. It spanned 12 months, with two waves of data collection. Using a mixed methods approach core trainees were required to complete survey with numerically rated items and open-ended questions pre- and post-intervention. Recruitment of core trainees and higher trainees was achieved through purposive sampling.

A 18-item survey was designed to enable quantitative analysis of training needs in Psychiatry and qualitative analysis of conceptions of mentorship. There were a total of 9 Likert questions and 1 open-ended question that enabled free text entry for qualitative analysis. A 23-item questionnaire was designed to evaluate Mentees response post-mentorship scheme.

**Results:** Pre-intervention: 75% identified career goals as an area that they would mostly likely value support with, closely followed by 68.3% reporting exam preparation, 31.3% reported support with e-Portfolio training and 25% with ARCP preparation as areas that they were hoping to get support with through mentorship.

Post-intervention: 66.7% reported improvement in competence in areas of difficulty which included:

55.6% improvement in clinical skills.

44.4% improvement in exam preparation.

66.7% improvement in diary management.

33.3% improvement in ARCP preparation.

44.4% improvement in e-portfolio training.

88.9% valued the presence of having to speak to someone as a useful aspect of the mentoring scheme and 44.4% reported recommending mentoring scheme to other trainees.

**Conclusion:** There is a breadth of evidence substantiating use of mentorship as a helpful tool in improving competence in doctors across different levels of their training. This finding was supported through a 12-month evaluation of the Mentorship scheme which appears to afford core trainees a cost-effective opportunity in improving training needs.

